# Genome-Wide Identification and Characterization of *AP2*/*ERF* Transcription Factor Family Genes in Oil Palm under Abiotic Stress Conditions

**DOI:** 10.3390/ijms22062821

**Published:** 2021-03-10

**Authors:** Lixia Zhou, Rajesh Yarra

**Affiliations:** Hainan Key Laboratory of Tropical Oil Crops Biology, Coconut Research Institute, Chinese Academy of Tropical Agricultural Sciences, Wenchang 571339, China; rajeshyarra@rediffmail.com

**Keywords:** AP2s, ERFs, stress responses, oil palm, qPCR

## Abstract

The *AP2/ERF* transcription factor family members play crucial roles in controlling plant growth and development, as well as responses to various abiotic stresses. Genome-wide identification and characterization of *AP2/ERF* genes has not yet been carried out in the oil palm genome. In the present work, we reported the occurrence of 172 *EgAP2/ERFs* (AP2, ERF, *RAV* & *Soloist* members) through genome-wide identification. Phylogenetic analysis was used to divide them into four groups, including: 34 AP2, 131 ERF, 5 *RAV*, and 2 *Soloist* gene family members. All 172 *AP2/ERF* members were unevenly distributed across 16 chromosomes of oil palm. Gene duplication analysis elucidated the tandem duplication of *AP2/ERF*s on chromosome blocks of the oil palm genome during evolution. Gene structure as well as conserved motif analysis demonstrated the conserved nature of intron/exon organization and motifs among the *AP2/ERF* genes. Several cis-regulatory elements—related to hormone, stress, and defense responses—were identified in the promoter regions of *AP2/ERF*s. Tissue-specific expression of 172 *AP2/ERF*s in five different tissues of oil palm was also revealed by heatmap analysis using the available transcriptome data. Finally, abiotic stress (salinity, cold & drought)-responsive *AP2/ERF*s in the oil palm genome were validated through qPCR analysis. Our study provided valuable information on oil palm *AP2/ERF* superfamily members and dissected their role in abiotic stress conditions.

## 1. Introduction

Transcription factors are one of the master regulators that control growth, development, and environmental stress responses in plants [1,2]. The APETALA2/ethylene-responsive element-binding factor (*AP2/ERF*) gene family is one of the largest and most prominent groups of transcription factors present in various plants [3,4]. The *AP2/ERF* transcription factorss comprise the highly conserved *AP2/ERF* DNA binding domains, with 60–70 amino acid residues. *AP2/ERF* TFs have been sorted into *AP2*, *ERF* (ethylene-responsive factor), *RAV* (related to ABI3/VP1), and *Soloist* families, depending on the number of conserved *AP2/ERF* domains and sequence similarities [4]. The AP2 family contains either single or double AP2 domains and plays a vital role in developmental processes [5]. The ERF & dehydration responsive element binding protein (DREB) families contain a single domain of AP2 and are involved in controlling various stress responses [6]. The *RAV* family consists of AP2 and B3 DNA binding domains, and is known to be involved in biotic and abiotic stress, especially regulated by two hormones (ethylene or brassinosteroids) [7,8]. The *Soloist* family is the smallest group that contains one AP2 domain and is majorly involved in salicylic acid-mediated defense against pathogens [9].

Based on whole-genome and EST sequence analyses, several researchers identified various numbers of *AP2/ERF* family members in the genomes of a wider range of plants, including: 147 in *Arabidopsis* [4,10]; 163 in rice [4,11]; 200 in poplar [12]; 132 in grapevine [13]; 131 in cucumber [14]; 218 in sugarcane [15]; 119 in Chinese jujube [16]; 98 in soybean [17]; 85 in tomato [18]; 117 in wheat [19]; 171 in cauliflower [20]; 134 in California poppy [21]; 193 in orchardgrass [22]; 174 in *Ammopiptanthus nanus* [23]; and 288 in sunflower [24]. Moreover, reports have described their prominent role in regulating abiotic stress responses (e.g., cold, salinity, drought, heat, and osmotic stress) in various plants [25,26,27,28,29,30]. Additional reports have demonstrated improved abiotic stress tolerance in transgenic plants via the expression of *AP2/ERF* TFs [31,32,33,34,35,36,37,38,39]. However, to date, no studies have elucidated the roles of *AP2/ERF* transcription factor members in the *Elaeis guineensis* genome under abiotic stress. Therefore, it iss important to identify the *AP2/ERF* TFs in *Elaeis guineensis* through a genome-wide identification approach.

Oil palm (*Elaeis guineensis*, Jacq.) is the most prominent oil-yielding tree in the world [40,41]. The productivity of oil palm plants is threatened by various environmental stresses, including cold, salinity, drought, and nutritional stress [42,43,44,45]. This can lead to substantial economic impact [46]. Therefore, it is necessary to develop oil palm varieties that tolerant to changing environmental conditions via molecular breeding strategies. To date, only a limited number of abiotic stress-responsive genes have been identified in the genome of oil palm. To the best of our knowledge, only WRKY [44] and MYB [42] transcription factors have been identified and partially characterized in response to various abiotic stresses. In view of this, we carried out genome-wide screening in order to identify other important transcription factors (such as *AP2/ERF* superfamily members) in oil palm. Additionally, expression analyses of identified oil palm *AP2/ERF* TFs under different abiotic stresses were performed.

In this study, a total of 172 *AP2/ERF* transcription family members were identified. In addition, we carried out analyses of the *AP2/ERF* gene structures, conserved domains, motif composition, phylogenetics, distribution (of 172 TFs on 16 chromosomes), gene duplication, and cis-acting elements. Finally, we investigated the relative expression of 18 *EgAP2/ERF* genes when oil palm plants were exposed to various abiotic stresses (salinity, drought, and cold) using real-time-PCR analysis. Our results shed light on an important aspect of identification and expression analysis of *AP2/ERF* transcription factor families under various abiotic stress conditions for the production of abiotic stress-resistant oil palm plants.

## 2. Results

### 2.1. Identification of EgAP2/ERF TFs

**Figure 1:**
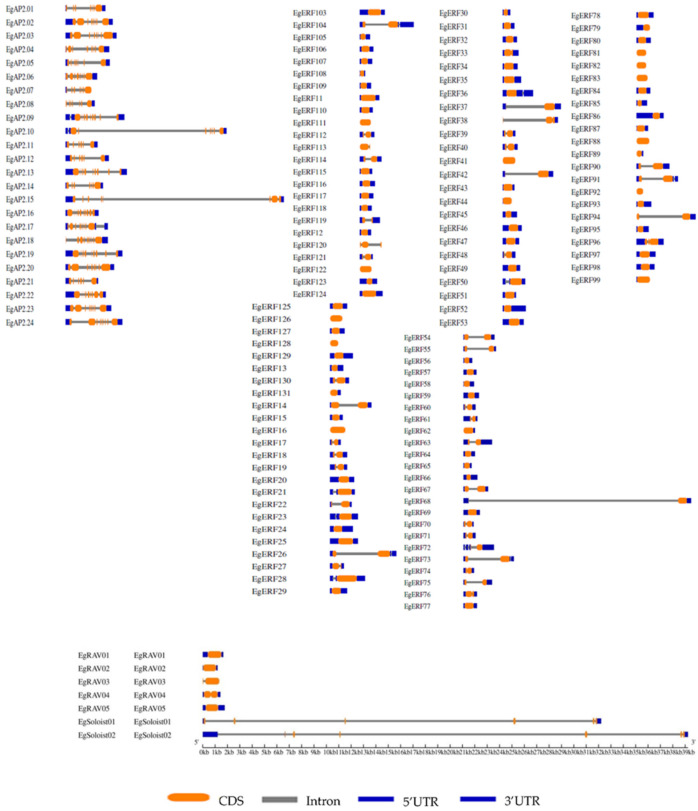
Intron-exon organization of 172 *AP2/ERF* transcription factors in the oil palm genome. Coding sequences (CDS) are represented by orange color blocks; 3′ & 5′ un-translated (UTR)regions are represented by grey color blocks; intron regions are represented by grey color blocks.

We identified a total of 172 *AP2/ERF* genes from the oil palm genome via a bioinformatic approach. The length of *EgAP2/ERF* genes ranged from 427–3373 bp (Supplementary Table S2). The Eg*AP2/ERF* proteins contained 124–663 of amino acids length (Supplementary Table S2). The identified *AP2/ERF* genes were categorized into four families (*AP2, ERF, RAV*, and *Soloist*) depending on the occurrence of conserved AP2 domains. Of the 172 *AP2/ERF* members identified, 34 of them belonged to the AP2 family, 131 of them belonged to the *ERF* family, 5 of them belonged to the *RAV* family, and only 2 belonged to the *Soloist* family (Supplementary Table S3). The identified number of Eg*AP2/ERF* superfamily gene members (172) was higher than that found in *Arabidopsis* and rice. Moreover, the number of introns among the four families varied; specifically, the 34 AP2 family members contained more introns (6–10) (Supplementary Table S2) (Figure 1). The ERF family members were mostly without introns, and a few of them contained 1–2 introns (Supplementary Table S2) (Figure 1). *RAV* family members *EgRAV*01, *EgRAV*02, and *EgRAV*05 were without introns. *EgRAV*03 and *EgRAV*04 had only one intron (Supplementary Table S2) (Figure 1). The remaining two *Soloist* family members (*EgSoloist*1 & *EgSoloist*2) had 6 and 7 introns, respectively (Supplementary Table S2) (Figure 1). The length of the individual introns differed in size and showed lengths in the range of 7–19,764 bp long. *EgRAV*04 had the smallest intron at 7 bp. The fourth intron of *EgSoloist*02 had 19,764 bp, which was followed by the intron of *EgERF*68 with 19,548 bp. Our subcellular localization prediction analysis revealed that the majority of *AP2, ERF, RAV,* and *Soloist* members localized to extracellular compartments, whereas a few members showed dual localization (Supplementary Table S2). However, some of the ERF members were strictly localized to the cytoplasmic region. Interestingly, none of the family members localized to a nuclear region (Supplementary Table S2).

### 2.2. Phylogenetic and Conserved Motif Analysis of EgAP2/ERF TFs

**Figure 2:**
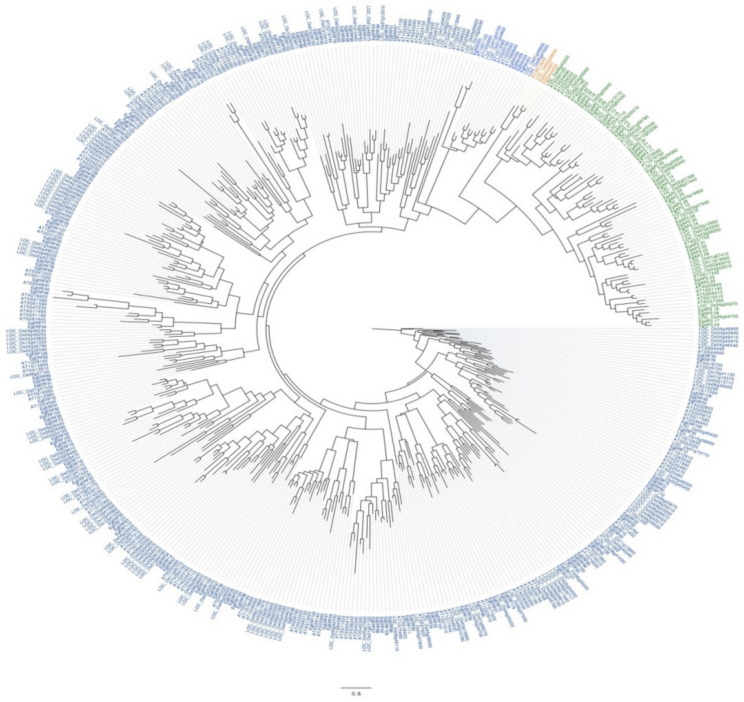
Phylogenetic analysis of *AP2/ERF* (AP2, ERF, *RAV*, and *Soloist*) family members in oil palm, *Arabidopsis,* and *Oryza sativa*. Each of the families from oil palm, *Arabidopsis*, and rice are grouped and represented in various colors (Navy blue, blue, orange, and green).

**Figure 3:**
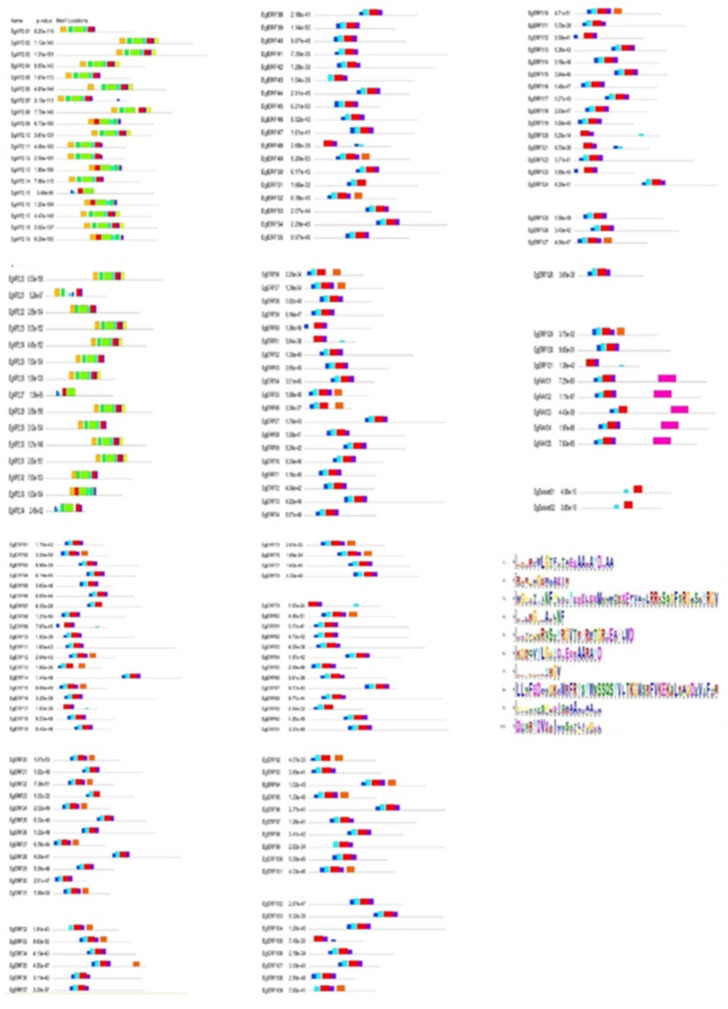
The architecture of conserved motifs in oil palm *AP2/ERF* proteins. Ten motifs were identified in *AP2/ERF* proteins using the MEME tool. Each motif in *AP2/ERF* proteins is represented with different colors. The abundance of each amino acid in every motif of oil palm *AP2/ERF* proteins is given in the sequence logo.

A phylogenetic tree was constructed using the deduced protein sequences of *AP2/ERF* genes from oil palm, rice, and *Arabidopsis.* The obtained phylogenetic tree grouped all of them into four main categories of *AP2/ERF* gene families (*AP2, ERF, RAV,* and *Soloist*) (Figure 2). Most of the sequences from oil palm, rice, and *Arabidopsis* grouped under *ERF* (followed by *AP2*) (Figure 2). A similar number of genes from oil palm, rice, and *Arabidopsis* clustered into the *RAV* family group. Moreover, the *Soloist* family had more numbers from oil palm (2) than *Arabidopsis* (1) and rice (1) (Figure 2). Furthermore, we analyzed the motif composition in each *AP2/ERF* family member using the online Multiple Expectation maximizations for Motif Elicitation (MEME) tool. Motif analysis revealed the occurrence of ten conserved motifs (1–10) from the identified oil palm *AP2/ERF* TFs (Figure 3). Results showed the existence of motif 4 and motif 5 in all four families of Eg*AP2/ERF* protein sequences (Figure 3). Motif 1, 2, 3, and 7 existed only in AP2 family members, whereas motif 6 was present in all except *Soloist* family members. Motif 10 was present only in *RAV* family members (Figure 3). The *Soloist* family members only contained two motifs (4 and 5). Motif 9 was restricted to ERF family members. The amino acid composition of each motif is illustrated in Figure 3.

### 2.3. Oil Palm AP2/ERF Genes Duplication Analysis

**Figure 4:**
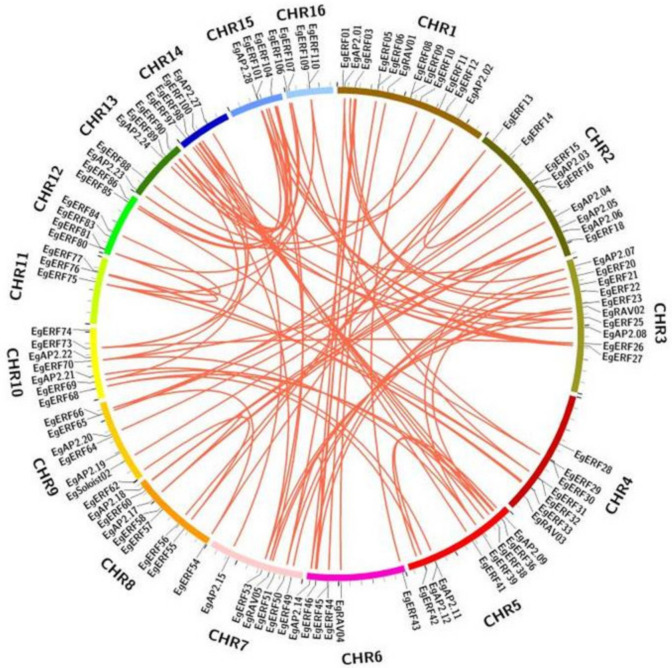
Schematic interpretation of AP2, ERF, *RAV*, and *Soloist* genes’ duplication in the oil palm genome. Each chromosome block is denoted with a different color. Red lines inside the schematic view denote the duplicated gene pairs of oil palm *AP2/ERF*s.

The gene duplication events analysis of *AP2/ERF* family members in the oil palm genome was examined via MCScanx and Circos software tools. The substitution ratios (ka/ks) (ka/ks > 1, positive selection; ka/ks = 1, neutral selection; ka/ks < 1, negative selection) were calculated in order to analyze the *AP2/ERF* gene duplication. The calculated Ka/Ks ratio values of all the *AP2/ERF* genes were found to be less than one (<1) (Supplementary Table S4). These results indicated that the evolution of oil palm *AP2/ERF* genes happened via strong purifying selection. Moreover, Eg*AP2/ERF* family members experienced tandem duplications across sixteen chromosomes during evolution (Figure 4). Furthermore, all 16 chromosomes of oil palm genome contained duplicated *AP2/ERF* family members (Figure 4). Chromosomes 3, 5, 13, 14, and 15 had more duplicated *AP2/ERF* gene pairs, whereas chromosomes 9 and 11 contained less. (Figure 4). These results demonstrate the prominent role of tandem duplication in the expansion of *AP2/ERF* gene family members in the oil palm genome.

### 2.4. Chromosomal Distribution of 172 EgAP2/ERF TFs

**Figure 5:**
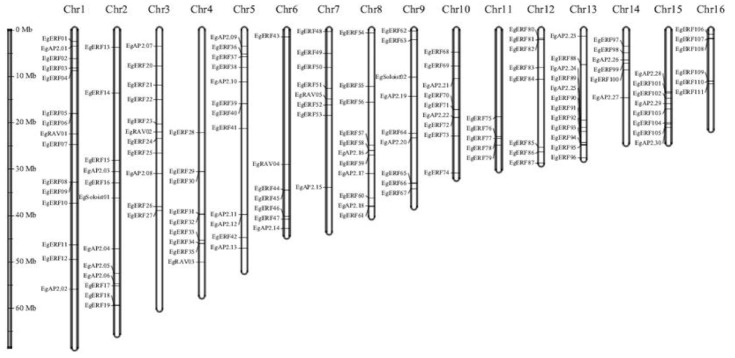
Chromosomal mapping of 172 oil palm *AP2/ERF*s. The length of the oil palm chromosomes is indicated on the vertical greyscale. The chromosome numbers (1–16) are indicated on the top of each chromosome.

All 172 identified Eg*AP2/ERF* genes were mapped to 16 chromosomes of oil palm. The distribution of Eg*AP2/ERF*s varied through 16 chromosomes of oil palm. Most (15) Eg*AP2/ERF*s were mapped on chromosome 1, followed by chromosome 2 (12) and chromosome 5 (12) (Figure 5). Chromosome 13 had the lowest number (2) of Eg*AP2/ERF*s (Figure 5). None of the AP2 family members were mapped to chromosomes 4, 11, 12, or 16; however, ERF family members were distributed on all 16 chromosomes of oil palm (Figure 5). Moreover, *RAV* family members were only mapped to chromosomes 3, 4, 6, 7 and 11—whereas *Soloist* family members were located only on chromosomes 2 and 9.

### 2.5. Analysis of Cis-Acting Elements in Promoter Regions of EgAP2/ERFs

**Figure 6:**
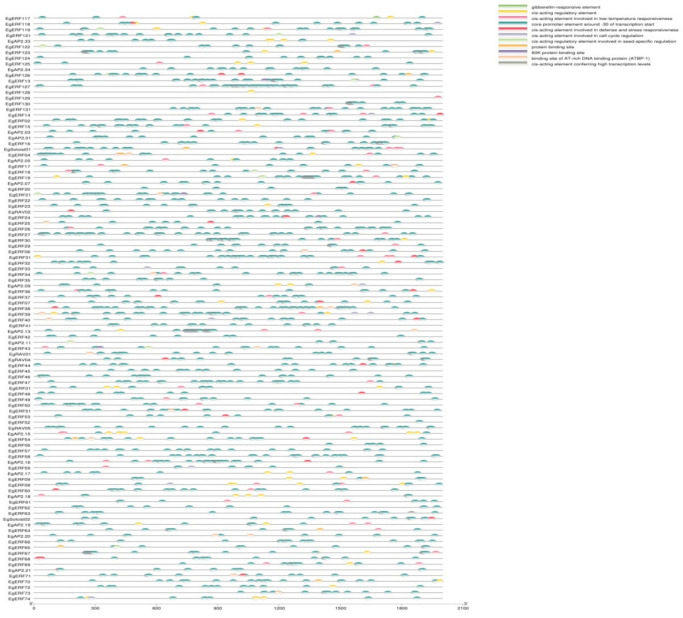
Schematic representations of cis-regulatory elements present in *AP2/ERF*s as investigated via the PlantCARE tool.

We analyzed the presence of various cis-acting elements located in the promoter regions of Eg*AP2/ERF*s using the PlantCare database. We found various cis-regulatory elements, related to the gibberellin response, defense response, stress response, and low temperature response. Additionally, those elements were involved in the regulation of cell cycle and seed-specific expression (Figure 6). However, the majority of Eg*AP2/ERF*s possessed cis-regulatory elements (CREs) responsible for the regulation of cell cycle and stress/defense responses (Figure 6). Most of the AP2 and ERF family members contained hormone, stress, and defense-responsive regulating elements. The *Soloist* family members contained stress-responsive elements, mostly confined to low-temperature stress-responsive elements (Figure 6). The *RAV* family members contained cell cycle regulating and stress-responsive elements but were mostly confined to CREs of cell cycle regulation (Figure 6).

### 2.6. Transcriptome Data-Based Tissue-Specific Expression Profiling of EgAP2/ERFs

**Figure 7:**
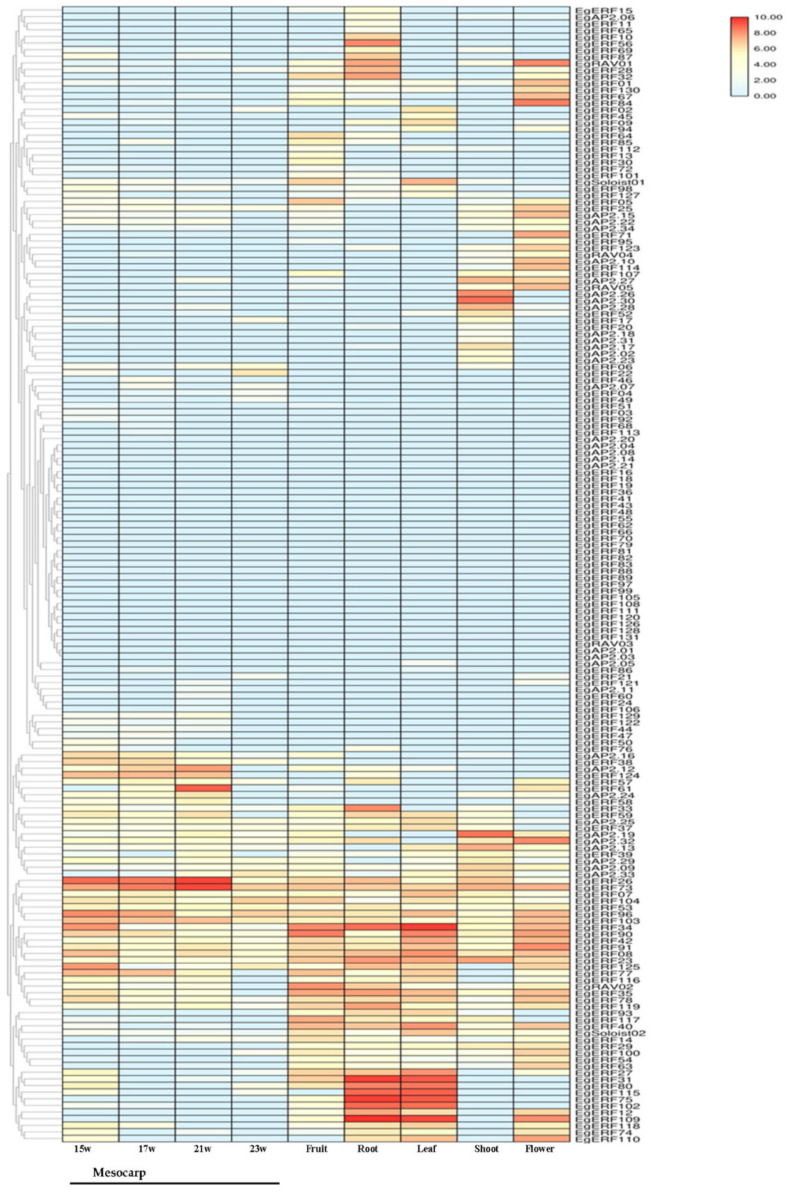
Heatmap for 172 Eg*AP2/ERF* gene expression in mesocarp (15, 17, 21 & 23 weeks old), root, shoot, leaf, flower, and fruit tissues of oil palm. Transcriptome data representation numbers (SRR190698, mesocarp (15 weeks); SRR190699, mesocarp (17 weeks); SRR190701, mesocarp (21 weeks); SRR190702, mesocarp (23 weeks); SRR851108, flower; SRR851067, fruit; SRR851096, leaf; SRR851071, root; SRR851103, shoot) are labeled in the bottom of the heatmap. The color scale represents the expression levels (low or high) of *AP2/ERF*s.

We analyzed the expression profiles of 172 Eg*AP2/ERF*s in six different tissues (leaf, root, fruit, flower, shoot, and mesocarp (15, 17, 21 and 23 weeks old)) of oil palm plants (unstressed) from the available transcriptome data (SRR851096, SRR851071, SRR851067, SRR851108, SRR851103, SRR190698, SRR190699, SRR190701 & SRR190702). Heatmap analysis showed that the majority of the genes were expressed at a lower level in oil palm tissues (Figure 7). A total of seven genes (*EgERF*27, *EgERF*31, *EgERF*34, *EgERF*75, *EgERF*80, *EgERF*102 & *EgERF*115) with the highest expressions were observed in both leaf and root tissues of oil palm (Figure 7). *EgERF*26, *EgERF*61, and *EgERF*73 showed higher expression in mesocarp tissues of 21 week-old oil palm (Figure 7). However, the majority of the gene expression was confined to the root, leaf, and fruit of oil palm. A higher number of genes showed a moderate level of expression in flowers. The majority of the AP2 members were down-regulated in oil palm tissues. The *RAV* members (such as *EgRAV*01 and *EgRAV*02) displayed the highest levels of expression in flower and fruit tissues, respectively (Figure 7). The *EgSoloist*02 gene expressed in leaf tissue of oil palm. Overall, it was indicated that ERF, AP2, *RAV*, and *Soloist* family members exhibited tissue-specific expression in oil palm (Figure 7).

### 2.7. EgAP2/ERF Genes’ Expression Analysis under Abiotic Stress

**Figure 8:**
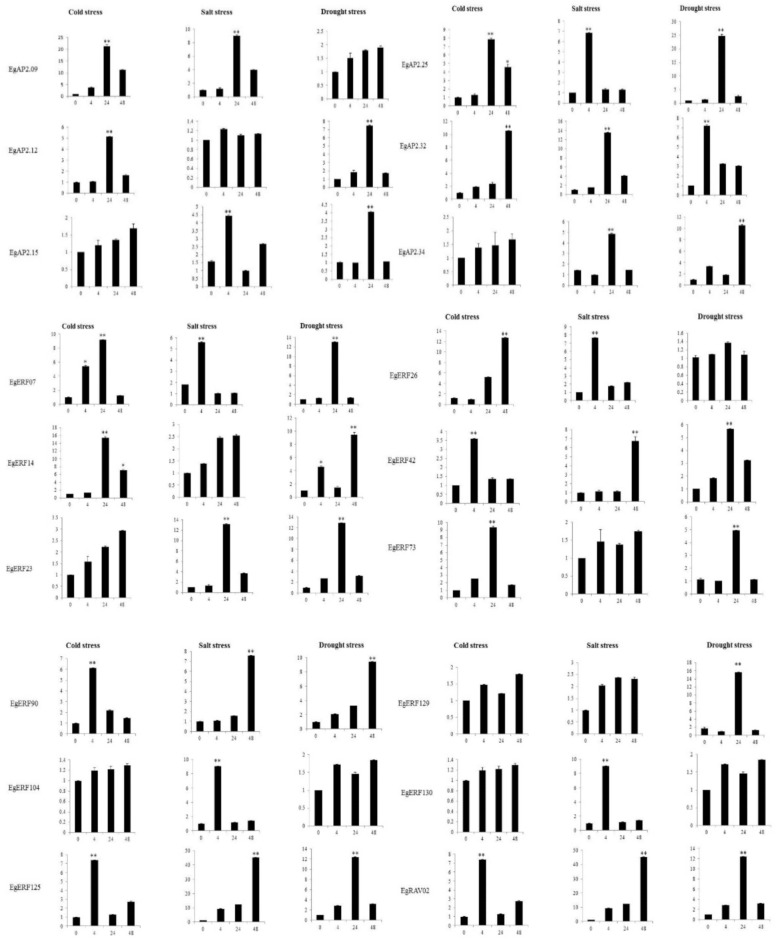
Real-time PCR analysis of 18 *AP2/ERF* transcription factors in response to cold, salt, and drought stress (0, 4, 24 & 48 h) conditions. *X*-axis represents the hours of stress exposure. *Y*-axis represents the relative expression of a transcription factor. Data represent the mean ± SE of three replicates. Asterisks represent significant difference at *p ≤* 0.05 (*) and *p ≤* 0.01 (**).

To further explore the responses of Eg*AP2/ERF* genes under abiotic stress conditions (salinity, drought, and cold), we examined the expression patterns of 18 Eg*AP2/ERF* genes (*EgAP2*.25, *EgAP2*32, *EgAP2* 34, *EgERF*07, *EgERF*14, *EgERF*23, *EgERF*26, *EgERF*42, *EgERF*73, *EgERF*90, *EgERF*104, *EgERF*125, *EgERF*129, *EgERF*130, and *EgRAV*02) from three different families via RT-qPCR analysis (Figure 8). Interestingly, all of the tested oil palm *AP2/ERF* gene family members were placed under salinity, drought, and cold stress conditions at a certain time (4 h, 24 h & 48 h). All of the salinity stress treatments (4 h, 24 h, 48 h) induced the expression of *EgERF*14, *EgERF*73, and *EgRAV*02 genes (Figure 8). The drought stress exposure at all time intervals (4 h, 24 h, and 48 h) induced the expression of *EgAP2*.09, *EgERF*26, *EgERF*90, and *EgERF*104 genes (Figure 8). The *EgAP2*.15, *EgAP2*.34, *EgERF*23, *EgERF*104, and *EgERF*130 genes showed increased expression under cold stress exposure (4 h, 24 h, and 48 h) (Figure 8). Our abiotic stress-responsive expression analysis results indicated the potential role of *AP2/ERF* family genes in regulating abiotic stress responses in oil palm.

## 3. Discussion

*AP2/ERF* superfamily genes act as vital regulators for governing various physiological and stress responses in a wide range of plants [25,26,27,28,29,30]. Few genome-wide studies have been carried out to identify *AP2/ERF* family members in plant genomes [4,10,11,13,14,15,16,17,18,19,20,21,22,23,24]. To date, no genome-wide studies have been conducted to explore the structure and role of *AP2/ERF* family genes in oil palm. Ours is thus the first report on the identification of *AP2/ERF* transcription factors in oil palm. In addition, we explored their role in various abiotic stress responses based on real-time PCR analysis.

The identified number (172) of *AP2/ERF*s in the *E. guineensis* genome was greater than the identified number of *AP2/ERF* members in various plants, but less than the number of *AP2/ERF* genes in poplar (200) [12], sugarcane (218) [15], orchardgrass(193) [23], *Ammopiptanthus nanus* (174) [22], and sunflower (288) [24] genomes. The oil palm *AP2/ERF* gene structural analysis revealed that a higher number of AP2 family members contained more introns (6–10) compared to ERF, *RAV* or *Soloist* family members. However, the occurrence of introns among all family members was very low. Our oil palm *AP2/ERF* gene structure analysis results were also consistent with other *AP2/ERF*s structural organizations observed in various plants [21,47,48,49]. Conserved motifs analysis was used to determine transcriptional activity, protein-protein interactions, and DNA-binding activity of TFs [50]. The conserved motif analysis of all 172 Eg*AP2/ERF* family members revealed the occurrence of 10 conserved motifs; those motifs might be related to specific functions shared among Eg*AP2/ERF* family members. Phylogenetic analysis of 172 oil palm *AP2/ERF* family members alongside *Arabidopsis* and rice indicated the conservation and diversification of AP2, ERF, *RAV*, and *Soloist* members among model-to-crop plants. Gene duplication events in plants commonly occur via tandem, whole-genome or segmental duplications [51]. Our study demonstrated the occurrence of tandem duplications in oil palm for the expansion of all *AP2/ERF* family members during evolution. These results also agreed with previous findings of the tandem duplications of *AP2/ERF* members in *Saccharum spontaneum* L. [15].

Several studies demonstrated that *AP2/ERF* family members were widely expressed in various tissues/organs for regulating the growth and development of plants [15,16,51]. The tissue-specific expression profiling data of genes is vital for elucidating the functional roles of genes. Our heatmaps analysis provided information on the expression of AP2, ERF, *RAV*, and *Soloist* members in the root, shoot, leaf, flower, and fruit mesocarp of oil palm plants. Each of the *AP2/ERF* family members (AP2, ERF, *RAV*, and *Soloist*) showed tissue-specific expression. However, the majority of them were expressed in all tissues, indicating their role in governing growth and developmental processes in oil palm plants.

Previous studies demonstrated the regulatory role of *AP2/ERF* members in response to various abiotic stress conditions [15,28,29,30,51,52]. Here, we examined the relative expression of 18 Eg*AP2/ERF* family members, including three AP2 genes (*EgAP2*.25, *EgAP2*32, *EgAP2* 34), eleven ERF genes (*EgERF*07, *EgERF*14, *EgERF*23, *EgERF*26, *EgERF*42, *EgERF*73, *EgERF*90, *EgERF*104, *EgERF*125, *EgERF*129, *EgERF*130) and one *RAV* family gene (*EgRAV*02) under salinity, cold, and drought stress conditions. All the tested transcription factors showed expression under all three abiotic stresses. The present study showed a higher expression of *EgERF*14, *EgERF*73, and *EgRAV*02 genes under salinity stress environments. Our results corroborated earlier reports on salt stress-induced ERF and *RAV* family members in various plants [15,52,53,54]. Higher expressions of *EgAP2*.09, *EgERF*26, *EgERF*90, and *EgERF*104 genes were also observed under drought stress conditions. The drought stress-induced AP2 and ERF family members were also reported in earlier studies and our results supported their findings [20,28,51,53]. In our study, cold stress induced the highest expressions of *EgAP2*.15, *EgAP2*.34, *EgERF*23, *EgERF*104, and *EgERF*130 genes; these results are in agreement with previous reports on cold-induced *AP2 and ERF* transcription factors [24,28,53,55]. These findings will be helpful in choosing potential *AP2/ERF* family members for the production of abiotic stress-tolerant oil palm varieties.

## 4. Materials and Methods

### 4.1. Database Search and Identification of AP2/ERF TFs in Oil Palm Genome

The genome sequences of African oil palm (*Elaeis guineensis*, Jacq.) were retrieved from the National Center for Biotechnology Information (NCBI) database. The known *AP2/ERF* sequences of *Arabidopsis thaliana* and *Oryza sativa* (ssp. Japonia and Indica) were downloaded from the Plntfdb (http://plntfdb.bio.uni-potsdam.de/v3.0/, accessed on 28 January 2021) and PlantTFDB (http://planttfdb.gao-lab.org/, accessed on 28 January 2021) databases and used as query sequences in the Basic Local Alignment Search Tool (BLAST) program to search for *AP2/ERF* genes in the oil palm genome. The conserved AP2 domains (PF00847) corresponding to *AP2/ERF* gene family members were obtained from the PFAM database (https://pfam.xfam.org/, accessed on 28 January 2021). The conserved domains database (CDD) online tool (https://www.ncbi.nlm.nih.gov/cdd/, accessed on 28 January 2021) was employed to verify the candidate gene sequences obtained from PFAM. A total of 172 Eg*AP2/ERF* genes were finally identified from the genome. The ExPASy proteomic website (https://web.expasy.org/compute_pi/, accessed on 28 January 2021) was employed to predict the molecular weight and isoelectric points of oil palm *AP2/ERF* proteins. Subcellular localization of all identified 172 Eg*AP2/ERF* proteins was predicted with the help of the CELLO tool (http://cello.life.nctu.edu.tw/, accessed on 28 January 2021).

### 4.2. EgAP2/ERF Gene Structure and Conserved Motif Analysis

Intron-exon organization of Eg*AP2/ERF*s was investigated by using the GSDS-2.0 online program (http://gsds.gao-lab.org/, accessed on 28 January 2021). The conserved motifs that existed in the identified *AP2/ERF* proteins were explored with the help of the Motif Elicitation (MEME) online program (http://meme-suite.org/tools/meme, accessed on 28 January 2021).

### 4.3. Phylogenetic Analysis, Chromosomal Distribution and Duplication Events Analysis of Oil Palm AP2/ERF Genes

The evolutionary relationships among *AP2/ERF* protein sequences of *Elaeis guineensis* (172 *AP2*/*ERF*s), *Arabidopsis thaliana,* and *Oryza sativa* (ssp. Japonia and Indica) were established by constructing a phylogenetic tree via the neighbor-joining method using MEGA 6.06 software. Various parameters (e.g., poison correction, pairwise deletion, and 1000 bootstrap replicates) were used to construct the phylogenetic tree. The chromosomal distribution of 172 Eg*AP2/ERF*s was investigated against the oil palm genome using th e TBtools software (https://github.com/CJ-Chen/TBtools, accessed on 28 January 2021). *AP2/ERF* gene duplication events were analyzed using the MCScanX tool (Multiple Collinearity Scan) with a set of parameters [56].

### 4.4. cis-Acting Elements Analysis of EgAP2/ERFs

The potential cis-regulatory elements of oil palm *AP2/ERF*s were checked by choosing 200 bp upstream of transcription start site TSS in each of *AP2/ERF* gene. We employed the PlantCARE online tool (http://bioinformatics.psb.ugent.be/webtools/plantcare/html/, accessed on 28 January 2021) to identify the cis-acting regulatory elements.

### 4.5. Source of Transcriptome Data and Tissue-Specific Expression Analysis of EgAP2/ERF TFs

The transcriptome data of oil palm tissues—including leaf, root, fruit, flower, shoot, and mesocarp (15, 17, 21, and 23 weeks old)—were downloaded from the SRA (Sequence Read Archive) database of the NCBI website. The transcript abundance of Eg*AP2/ERF*s in different tissues was calculated with RPKM (reads per kilobase per million mapped reads) values. The heatmap tool was used to generate a heatmap of all 172 Eg*AP2/ERF*s.

### 4.6. Oil Palm Plant Materials and Stress Exposure

The African oil palm *(Elaeis guineensis* Jacq.) plantlets were raised in greenhouses (27 °C/temperature; 16 h/light; 8 h/darkness; humidity of about 50–60%) at Coconut Research Institute, CATAS, Wenchang, China. Healthy oil palm plantlets of the same age (6 months old) were chosen to undergo abiotic stress treatments (cold, salt, and drought). Before stress exposure, all plants were shifted to a growth chamber at 27 °C for one day. Each abiotic stress treatment (cold or drought or salinity) was done after 0 h (control), 4 h, 24 h, and 48 h, using different seedlings (9 for each treatment). Oil palm seedlings were exposed to cold stress exposure at 8 °C. Drought stress condition for the oil palm seedlings was established after reaching 20% water content in the soil. Salinity stress was induced by immersing the roots of oil palm seedlings in 300 mmol/L concentration of NaCl solution. At different time intervals (0, 4, 24 & 48 h) of the above stress exposures, the spear leaves were collected and immediately frozen in liquid nitrogen for further RT-qPCR analysis. In addition, oil palm seedlings for control experiments were maintained under light (16 h) and darkness (8 h) at 27 °C. All the stress experiments used three biological replicates and were repeated three times.

### 4.7. Quantitative Real-Time PCR (RT-qPCR) Analysis of EgAP2/ERFs

Total RNA was extracted from leaf samples collected before and after stress treatments, following the previously reported protocol (Iqbal et al., 2009). The isolated RNA (~2 µg) was then reverse transcribed to cDNA using a MightyScript Plus first-strand cDNA synthesis kit for analyzing the relative expression of Eg*AP2/ERF*s under abiotic stress conditions. The RT-qPCR amplifications were executed in a Mastercycler^®^ ep realplex Real-time PCR System in 384-well optical plates. A high-throughput qPCR primer designing tool (Quantprime (https://quantprime.mpimp-golm.mpg.de/, accessed on 28 January 2021)) was used to design *AP2/ERF* gene-specific primers. The thermal conditions for amplification reactions were set at 95 °C/5 s, 58 °C/15 s, and 68 °C/20 s. All the amplifications were done with three biological and three technical repeats. The 2^−ΔΔCt^ method was used to calculate the relative expression of all tested *AP2/ERF* genes. The list of qPCR primers used in this study is provided in Supplementary Table S1.

### 4.8. Statistical Analysis

The experiments were performed with three technical and biological replications. One-way analysis of variance (ANOVA) was used to determine the statistical significance at the *p* ≤ 0.05 and *p* ≤ 0.01 levels. Asterisks represent a significant difference at *p* ≤ 0.05 (*) and *p* ≤ 0.01 (**).

## 5. Conclusions

A total of 172 *AP2/ERF* family TFs were identified in the African oil palm genome through bioinformatic analysis. Our bioinformatic approach revealed the *AP2/ERF* genes’ structural organization, distribution across 16 chromosomes, motif conservation among all the family members, and duplication events of all AP2, ERF, *RAV*, and *Soloist* gene family members. Furthermore, we predicted subcellular localization and analyzed the cis-acting regulatory elements in promoter regions and tissue-specific expression of oil palm *AP2/ERF* family members. Additionally, our experimental approach validated the role *AP2/ERF* TFs play in various abiotic stress conditions. Overall, our bioinformatic and experimental approaches have provided valuable information on oil palm *AP2/ERF* genes—information that can be used for the development of abiotic stress-tolerant oil palm varieties in the near future.

## Data Availability

The data presented in this study are available in supplementary file.

## Supplementary Materials

The following are available online at https://www.mdpi.com/1422-0067/22/6/2821/s1, Table S1; Table S2; Table S3; Table S4.
